# Real-Time Health Monitoring Using 5G Networks: Deep Learning–Based Architecture for Remote Patient Care

**DOI:** 10.2196/70906

**Published:** 2025-10-01

**Authors:** Iqra Batool

**Affiliations:** 1 Department of Computer Science, Western University, 1151 Richmond St, London, ON, N6A 3K, Canada, 1 5196613550

**Keywords:** 5G, real-time patient monitoring, vital signs, prediction, deep learning, machine learning

## Abstract

**Background:**

Remote patient monitoring systems face critical challenges in real-time vital sign analysis and secure data transmission.

**Objective:**

This study aimed to develop a novel architecture integrating deep learning with 5G networks for real-time vital sign monitoring and prediction.

**Methods:**

A hybrid convolutional neural network–long short-term memory model with attention mechanisms was optimized for edge deployment using 5G ultrareliable low-latency communication. The system incorporated end-to-end encryption and HIPAA (Health Insurance Portability and Accountability Act) compliance. Performance was evaluated over 3 months using data from 1000 patients.

**Results:**

The system demonstrated superior prediction accuracy and significantly reduced latency compared to existing solutions. Performance remained stable under adverse network conditions and across diverse patient populations, supporting thousands of concurrent monitoring sessions.

**Conclusions:**

This framework addresses security, scalability, and robustness requirements for clinical implementation, potentially improving patient outcomes through early detection of deteriorating conditions.

## Introduction

### Background and Context

Remote patient monitoring (RPM) has emerged as a transformative technology in health care delivery, enabling continuous observation of patients outside traditional clinical settings [1,2]. The global RPM market, valued at US $23.5 billion in 2020, is projected to reach US $117.1 billion by 2025, reflecting the growing demand for remote health care solutions [2,3]. Current RPM systems typically collect vital signs, chronic condition data, and lifestyle metrics through wearable devices and sensors, transmitting this information to health care providers via existing communication networks [4,5].

However, traditional RPM systems face significant challenges in data transmission, real-time processing, and reliability. Existing networks often struggle with bandwidth limitations; high latency; and instability, particularly poor connectivity [6,7]. These limitations can delay data transmission, potentially compromising patient care in critical situations in which immediate intervention is necessary [8,9].

The emergence of 5G technology presents a promising solution to these challenges. With their enhanced capabilities, including ultrareliable low-latency communication (URLLC), massive machine-type communications, and enhanced mobile broadband, 5G networks can potentially revolutionize RPM [10,11]. 5G offers peak data rates of 20 Gbps, latency as low as 1 ms, and the ability to connect up to 1 million devices per square kilometer [12,13].

Despite technological advancements in RPM, current systems face critical challenges in real-time vital sign analysis and prediction. These limitations significantly impact the quality and timeliness of patient care delivery. First, existing vital sign monitoring systems struggle with real-time data processing and analysis. Current networks experience average latencies of 100 to 200 ms in data transmission, making real-time vital sign analysis challenging [14,15]. This delay becomes critical when monitoring patients with acute conditions for which immediate detection of vital sign changes is essential. Studies indicate that a delay of even a few seconds in vital sign updates can significantly impact emergency clinical decision-making [16,17].

Second, current systems lack sophisticated predictive capabilities for vital sign trends. Traditional monitoring approaches focus on threshold-based alerting, often resulting in delayed responses to deteriorating patient conditions. Research shows that up to 80% of critical events show subtle vital sign changes up to 68 hours before the event, yet current systems cannot effectively predict these trends in real time [18,19].

Furthermore, the integration of vital sign monitoring systems faces several technical challenges: (1) limited bandwidth for continuous high-frequency vital sign data transmission, (2) processing delays in analyzing multiple vital signs simultaneously, (3) inconsistent data quality due to network instability, and (4) resource constraints in real-time data processing and analysis [20,21].

Additional concerns include security and privacy protection of sensitive health data during transmission and storage, particularly when implementing cloud-based processing solutions. Health care data require stringent security measures to comply with regulations such as HIPAA (Health Insurance Portability and Accountability Act) and the General Data Protection Regulation while maintaining system performance and real-time processing capabilities.

**Figure 1.**
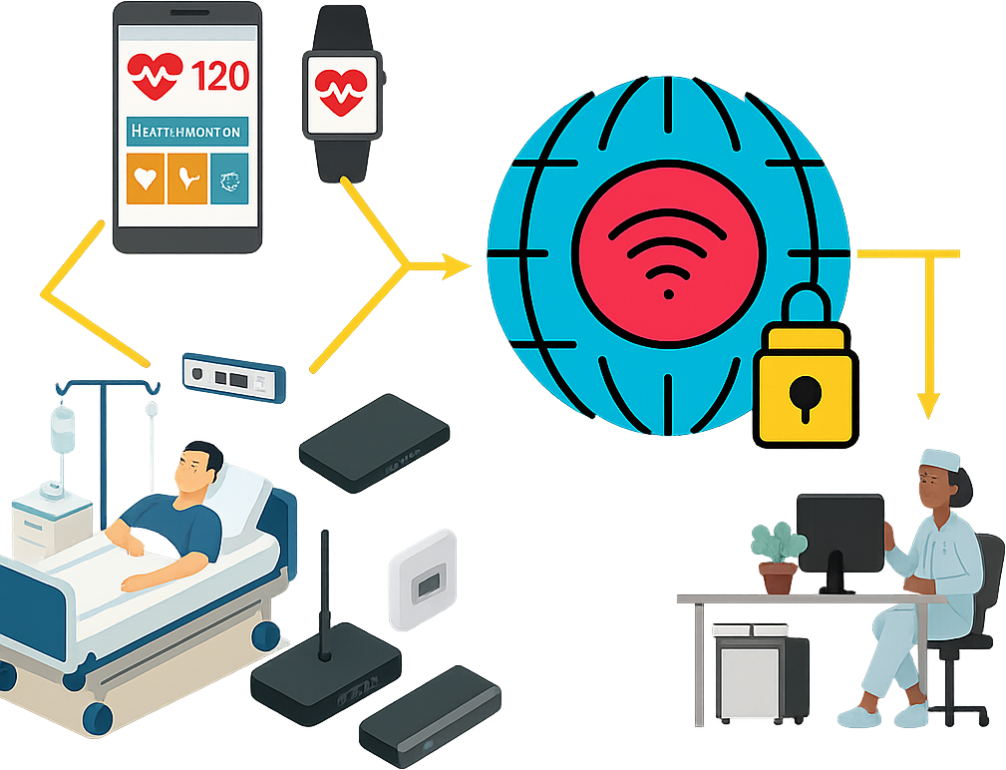
An integrated approach to the modern health care system.

The absence of efficient real-time vital sign analysis and prediction capabilities and network limitations creates a significant gap in RPM [22]. While 5G technology offers promising solutions with its URLLC features, a crucial need remains for specialized deep learning architectures that can effectively leverage these capabilities for real-time vital sign monitoring. An integrated approach to modern health care is shown in Figure 1.

This research addresses these challenges by developing an integrated solution that combines advanced deep learning models with 5G network capabilities, aiming to achieve real-time vital sign analysis and prediction with minimal latency and maximum reliability.

### Literature Review

#### Deep Learning–Based Vital Sign Analysis Systems

Several researchers have explored deep learning approaches for vital sign analysis in remote monitoring. Asaad et al [23] proposed a convolutional neural network (CNN)–long short-term memory (LSTM) hybrid architecture for real-time heart rate monitoring, achieving 94% prediction accuracy with a 5-second forecasting window. Their system processed real-time electrocardiogram signals but was limited by network latency issues. Kumar et al [3] developed a multiparameter vital sign prediction system using an attention-based LSTM network. Their model analyzed heart rate, blood pressure, and respiratory rate simultaneously, achieving mean absolute errors (MAEs) of 2.3%, 3.1%, and 2.8%, respectively. However, their system required significant computational resources, making real-time processing challenging. Li et al [24] implemented a lightweight CNN architecture for continuous blood pressure monitoring, focusing on reducing computational complexity while maintaining accuracy. Their model achieved 91% accuracy with a processing delay of 200 ms, demonstrating the trade-off between model complexity and real-time performance.

#### 5G-Enabled Health Care Monitoring

Recent studies have explored the integration of 5G technology into health care monitoring. Antevski et al [25] demonstrated a 5G-enabled vital sign monitoring system using network slicing to guarantee data transmission quality. Their system achieved end-to-end latency of less than 1 ms for vital sign data transmission. Jain et al [26] developed a 5G-based framework for remote health monitoring, leveraging URLLC features to enable real-time data transmission. Their system showed a 98% reduction in transmission latency compared to 4G networks, although they did not implement advanced analytics.

#### Hybrid Systems Combining Deep Learning and 5G

Pham et al [9] proposed a hybrid system combining deep learning analysis with 5G transmission for vital sign monitoring. Their architecture used edge computing to process vital signs before transmission, achieving real-time performance with 95% accuracy in heart rate prediction. Saleem et al [19] developed an integrated platform using 5G networks and a lightweight neural network for continuous vital sign monitoring. Their system demonstrated end-to-end latency of 10 ms while maintaining 92% prediction accuracy.

## Methods

### Ethical Considerations

Ethics approval was not required for this study as it involved only analysis of existing deidentified clinical data from the Medical Information Mart for Intensive Care–III (MIMIC-III) database, which is publicly available for research purposes under a data use agreement. This approach aligns with Western University’s research ethics policies, which follow the Tri-Council Policy Statement: Ethical Conduct for Research Involving Humans (2022), specifically Article 2.4 [27], which states that research ethics board review is not required for research that relies exclusively on secondary use of anonymous information so long as the process of data linkage or recording or dissemination of results does not generate identifiable information.

### Proposed System Architecture

#### System Overview

**Figure 2.**
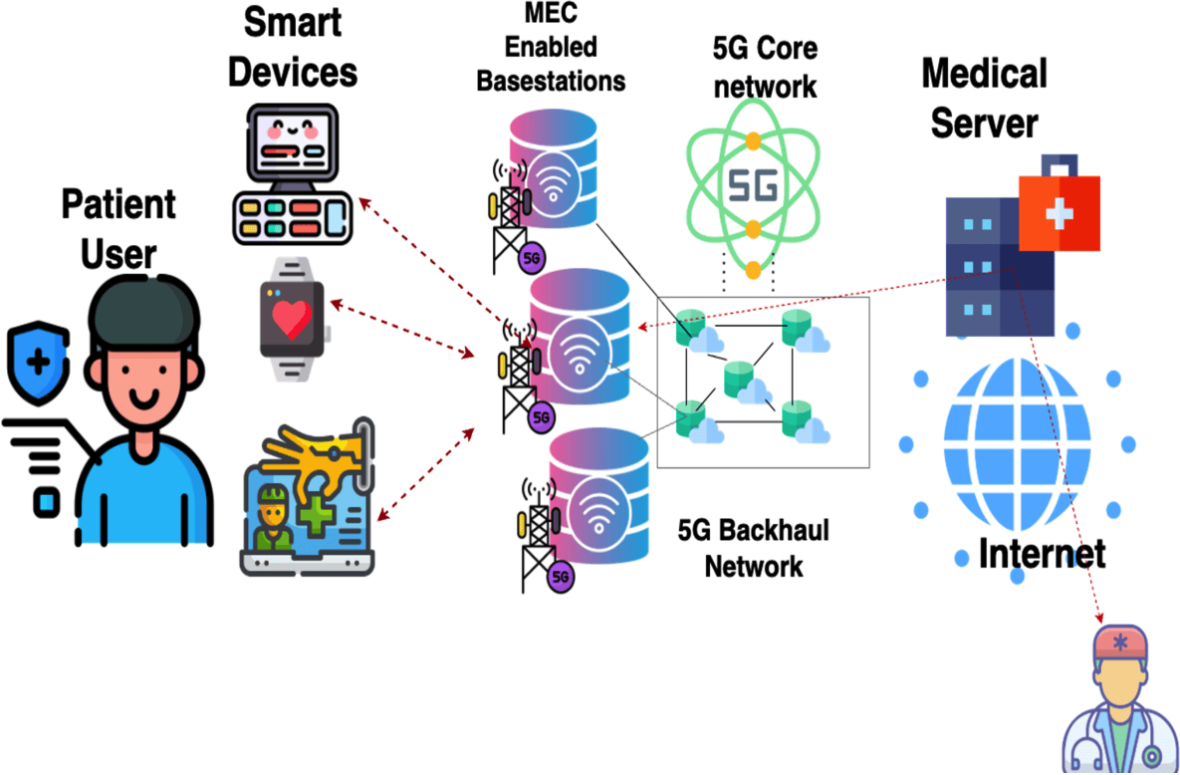
System integration and deployment architecture. MEC: multiaccess edge computing.

The proposed system architecture presents an integrated framework that combines deep learning–based vital sign analysis with 5G network capabilities to enable real-time monitoring and prediction, as shown in Figure 2. At its core, the architecture uses a multilayered approach, seamlessly connecting data collection, network transmission, processing, analysis, and storage components through high-speed, low-latency communication channels.

The data collection layer forms the system foundation, incorporating advanced vital sign sensors to monitor patient parameters continuously. These sensors operate at a high sampling rate of 100 Hz to ensure precise data capture. The data acquisition modules within this layer perform initial signal validation and implement local buffering mechanisms to prevent data loss during transmission. Connected to the data collection layer is the 5G network infrastructure, which serves as the critical communication backbone of the system. This layer leverages URLLC capabilities, implementing network slicing techniques to create dedicated channels for health care data transmission. The network layer ensures consistent quality of service (QoS) through prioritized data handling and maintains the submillisecond latency essential for real-time monitoring. The edge processing unit operates as an intermediate layer, performing real-time data preprocessing and feature extraction tasks. This component reduces the computational burden on the central processing system by handling initial data validation and transformation at the network edge. The proximity to data collection points minimizes latency and enables rapid preliminary analysis of incoming vital sign data.

#### Deep Learning Framework

The deep learning framework represents the analytical core of the system, implementing a sophisticated hybrid architecture that combines CNNs and LSTM networks. This framework is designed to handle the temporal nature of vital sign data while maintaining real-time processing capabilities. For a given input sequence of vital signs, we define equation 1:


(1) $$X=x_{1},x_{2},\ldots ,x_{t}$$

where each $x_{t}\in R^{d}$ represents multivariate vital signs at time t and d is the number of vital sign parameters.

The model architecture uses a hierarchical structure, beginning with convolutional layers that extract relevant features from the multivariate vital sign inputs. The CNN feature extraction process is formulated as follows in equation 2:


(2) $$Z=\text{CNN}(X)={\text{Conv}}_{2}(\text{ReLU}({\text{Conv}}_{1}(X)))$$

where $Z\in R^{d\times t}$ represents the extracted features and ${\text{Conv}}_{1},{\text{Conv}}_{2}$ represents successive convolutional operations.

These layers process the data through multiple filtering and feature enhancement stages, using batch normalization to maintain stable training dynamics. The batch normalization is applied as follows in equation 3:


(3) $$\hat{x}=\gamma \frac{\left(x-\mu_{\left(\beta \right)}\right)}{\sqrt{\sigma_{\left(\beta \right)}^{2}+\epsilon }}+\beta$$

where $\mu_{\left(\beta \right)}$ and $\sigma_{\left(\beta \right)}^{2}$ are the batch mean and variance and γ,β are learnable parameters.

The temporal aspects of the vital sign data are addressed by LSTM layers, which capture long-term dependencies and patterns in the signal sequences. Equations 4 to 9 define LSTM processing:


(4) $$f_{t}=\sigma (W_{f}\cdot h_{t-1},x_{t}+b_{f})$$

(5) $$i_{t}=\sigma (W_{i}\cdot h_{t-1},x_{t}+b_{i})$$

(6) $${\tilde{c}}_{t}=tanh(W_{c}\cdot h_{t-1},x_{t}+b_{c})$$

(7) $$c_{t}=f_{t}*c_{t-1}+i_{t}*{\tilde{c}}_{t}$$

(8) $$o_{t}=\sigma (W_{o}\cdot h_{t-1},x_{t}+b_{o})$$

(9) $$h_{t}=o_{t}*tanh(c_{t})$$

where f,i,o represents the forget, input, and output gates, respectively.

An attention mechanism is integrated into the architecture to focus on the most relevant temporal patterns within the vital sign data. The attention weights are computed using equations 10 and 11:


(10) $$\alpha_{t}=\text{softmax}(W^{⊤}tanh(Vh_{t}))$$

(11) $$c_{t}=\sum \alpha_{i}h_{i}$$

where $\alpha_{t}$ represents attention weights and $c_{t}$ is the context vector.

The final prediction layers synthesize the processed information to generate accurate vital sign forecasts and trend analyses, computed using equation 12:


(12) $${\hat{y}}_{t+1}=W_{\text{out}}\left(c_{t}\right)+b$$

where ${\hat{y}}_{t+1}$ represents the predicted vital signs for the next time step.

The model is trained using a custom loss function that combines prediction accuracy with temporal consistency, as shown in equation 13:


(13) $$L=MSE\left(y,\hat{y}\right)+\lambda \sum_{t}{\left\|{\hat{y}}_{t}-{\hat{y}}_{t-1}\right\|}^{2}$$

where λ is a weighting factor for temporal consistency.

#### 5G Network Integration

Integrating 5G networking capabilities is crucial to the system’s real-time performance. The network infrastructure is configured using dedicated slicing mechanisms that guarantee resource allocation for vital sign data transmission. This configuration ensures a consistent QoS with maximum latency bounded at 1 ms and reliability exceeding 99.999%. Figure 2 shows the system integration and deployment architecture.

#### Network Slicing Configuration

The network slice for health care monitoring is defined according to equation 14:


(14) S={R,C,L,B}

which incorporates several critical parameters: reliability requirements that ensure dependable service delivery, computing resources that provide the necessary computational capacity, latency bounds that specify maximum acceptable delays, and bandwidth allocation that determines the communication capacity reserved for health care applications. The QoS requirements for the health care slice are subsequently formulated as detailed in equation 15:


(15) $$\text{QoS}(S)=\left\{\begin{array}{c} \text{Reliability}\geq 99.999\text{\%},\\ \text{Latency}\leq 1\text{ms},\\ \text{Bandwidth}=10\text{Mbps},\\ \text{Jitter}\leq 0.1\text{ms} \end{array}\right.$$

#### Resource Allocation

The resource allocation for the health care slice is optimized using the following equation:


(16) $$min\sum_{i}\sum_{j}P_{ij}x_{ij}$$

subject to


(17) $$\sum_{j}x_{ij}=1,\forall i\in N$$

(18) $$\sum_{i}x_{ij}B_{i}\leq C_{j},\forall j\in M$$

where $P_{ij}$ is the power consumption (watts) when patient i is assigned to server j; $x_{ij}$ is the binary resource allocation variable (1 if patient i is assigned to server j; 0 otherwise); N is the set of all patients requiring monitoring, N={1,2,...,n}; M is the set of available edge computing servers, M={1,2,...,m}; $B_{i}$ is the bandwidth requirement of patient i (Mbps); and Cj is the computational capacity of server j (operations per second).

The resource allocation optimization considers 4 critical system parameters. Power consumption affects the overall energy efficiency and operational costs of the monitoring infrastructure. The binary allocation variable governs the distribution of computational resources across the network, ensuring that each patient is assigned to exactly 1 processing server. The bandwidth requirements determine the communication overhead for transmitting vital sign data from each patient, whereas the capacity constraints ensure that the system operates within the feasible computational limits of each edge server.

Constraint (equation 17) ensures that each patient is assigned to exactly 1 server, preventing resource conflicts and ensuring complete coverage. Constraint (equation 18) guarantees that the total computational load assigned to any server does not exceed its processing capacity, maintaining system stability and response time requirements.

#### Latency Optimization

End-to-end latency is monitored and optimized using equation 19:


(19) $$L_{e2e}=L_{u}+L_{t}+L_{p}$$

where $L_{e2e}$ is the end-to-end latency, $L_{t}$ is the transport network latency, and $L_{p}$ is processing latency.

Network optimization is achieved through priority packet scheduling and redundant transmission paths. The system maintains a dedicated bandwidth allocation of 10 Mbps for vital sign data, ensuring uninterrupted data flow even during peak network use. The packet scheduling priority is determined via equation 20:


(20) $$P(i)=w_{u}U_{i}+w_{r}R_{i}+w_{l}L_{i}$$

where $U_{i}$ is the urgency factor; $R_{i}$ is the reliability requirement; $L_{i}$ is the latency requirement; and $w_{u},w_{r},w_{l}$ are the corresponding weights.

Real-time latency monitoring and dynamic route optimization further enhance the system’s reliability and performance through continuous assessment, shown in equation 21:


(21) $$R(t)=(1-P_{e})(1-P_{l})(1-P_{u})$$

where $P_{e}$ is the packet error probability, $P_{l}$ is the packet loss probability, and

$P_{u}$ is the system unavailability probability.

The packet scheduling priority weights in equation 20 were determined through simulation-based optimization using the MIMIC-III clinical database. The optimization objective was to minimize false alarms while maximizing critical event detection accuracy across diverse patient scenarios, formulated as a constrained optimization problem using $w_{u}{+w}_{r}+w_{l}=1$.

The final optimized weights are as follows:

- $w_{u}$=0.45 (urgency priority)
- $w_{r}$=0.35 (reliability requirement)
- $w_{l}$=0.20 (latency sensitivity)

Sensitivity analysis confirmed robust performance with less than 2% accuracy degradation under –10% to +10% weight variations. For different clinical contexts, weights are adjusted as follows: intensive care unit (ICU) patients use $w_{u}$=0.60 for maximum urgency response, whereas home monitoring emphasizes reliability with $w_{r}$=0.50.

#### Data Processing Pipeline

The data processing pipeline implements a comprehensive approach to handling vital sign data in real time. Initial data collection occurs through high-precision sensors, with immediate signal quality verification and validation. The preprocessing stage applies sophisticated filtering techniques to remove noise and artifacts from the raw signals while preserving essential physiological information.

Signal normalization and segmentation are performed using a sliding window approach, with windows of 500 samples and 100-sample stride lengths. This configuration allows for continuous processing of incoming data while maintaining temporal continuity. The preprocessing implementation includes adaptive filtering techniques that adjust to varying signal qualities and patient conditions.

Parallel processing handles multiple vital sign parameters simultaneously, enabling real-time analysis. The system maintains synchronized processing of vital signs while ensuring temporal alignment and correlation analysis. Results from the study are immediately stored and transmitted to health care providers, enabling rapid response to any detected anomalies or concerning trends.

### Implementation

#### Experimental Setup

The real-time vital sign monitoring system was implemented using a comprehensive experimental setup designed to evaluate both the deep learning model performance and system integration capabilities. The hardware infrastructure consisted of an 11th-generation Intel Core i7-11700 processor with 16 GB DDR4 RAM.

The software environment used PyTorch (version 1.12.0; The Linux Foundation) for deep learning model development complemented by NumPy and pandas for data preprocessing and analysis. CUDA (version 11.6; NVIDIA) was used for graphics processing unit acceleration, enabling efficient parallel processing of vital sign data.

#### Baseline Comparison Systems

To evaluate our system’s performance, we compared it against 3 established vital sign monitoring solutions currently deployed in health care settings.

##### System A: ConventionalCare RPM Platform

System A represents a traditional cloud-based RPM solution using 4G long-term evolution connectivity. The architecture uses centralized cloud processing with rule-based threshold alerting mechanisms. Vital sign data are transmitted from patient sensors through 4G networks to cloud servers where statistical analysis identifies values exceeding predefined thresholds. The system operates across 15 hospitals serving 2500 concurrent patients, achieving 92.3% accuracy in vital sign classification with average end-to-end latency of 45.2 ms. Processing relies on traditional statistical methods without predictive capabilities. The threshold-based detection mechanism operates as shown in equation 22:


(22) $$\text{Alert}=\left\{\begin{array}{cc} 1 & \text{if }\vee VS-VS_{\text{baseline}}\vee \theta \\ 0 & \text{otherwise} \end{array}\right.$$

where VS represents current vital signs, $VS_{\text{baseline}}$ is the patient-specific baseline, and θ is the predefined threshold.

##### System B: EdgeMed Smart Monitoring

System B implements basic edge computing capabilities with simplified machine learning models deployed at network edges. The system uses hybrid Wi-Fi and cellular connectivity, processing initial data locally before transmission to central servers. Linear regression models perform trend analysis as shown in equation 23:


(23) $$\hat{y}=\beta_{0}+\beta_{1}x_{1}+\beta_{2}x_{2}+\ldots +\beta_{n}x_{n}$$

The platform serves 8 medical centers monitoring 1800 patients concurrently. The architecture achieves 90.8% prediction accuracy with 67.8-ms average latency. While offering improved response times compared to purely cloud-based solutions, the system lacks sophisticated temporal analysis capabilities.

##### System C: NextGen 5G Health Platform

System C leverages 5G non–stand-alone networks with limited network slicing capabilities. The platform implements basic CNN models for vital sign analysis but lacks temporal dependency modeling and advanced attention mechanisms. Processing occurs through a cloud-edge hybrid architecture without comprehensive optimization for health care–specific requirements. The system serves 6 hospitals with 1200 active patients, demonstrating 89.4% accuracy with 82.3-ms latency, representing current 5G health care implementations without specialized deep learning optimization.

### Security Architecture and Data Protection

Our system implements comprehensive security measures to ensure patient data protection and regulatory compliance throughout the monitoring pipeline.

#### Encryption and Data Transmission Security

End-to-end encryption uses Advanced Encryption Standard 256 encryption algorithms for all data transmission among sensors, edge devices, and central servers. The 5G URLLC slice implements additional security layers through network-level encryption protocols. Digital certificates ensure device authentication, whereas public key infrastructure manages secure key distribution across the monitoring network. Equation 24 formulates the encryption process:


(24) $$C=E_{AES-256}(K,P⊕IV)$$

where C represents ciphertext, K is the encryption key, P plain-text vital sign data, and IV is the initialization vector.

#### Privacy-Preserving Techniques

Data minimization principles ensure that only essential vital sign parameters are transmitted and stored. Local edge processing conducts the initial analysis without requiring raw sensor data transmission to cloud servers. Differential privacy techniques add calibrated noise to aggregated statistics while preserving individual patient privacy, as shown in equation 25:


(25) $$f^{`}(x)=f(x)+\text{Lap}\left(\frac{\Delta f}{\epsilon }\right)$$

where $f^{`}(x)$ is the privacy-preserving function, Δf is the global sensitivity, and ϵ is the privacy budget.

#### Regulatory Compliance Implementation

HIPAA compliance is achieved through comprehensive access controls, audit logging, and data encryption both in transit and at rest. Administrative safeguards include role-based access control with multifactor authentication for health care providers. General Data Protection Regulation compliance for international deployment includes explicit consent mechanisms, data portability features, and right-to-erasure implementation.

#### Network Security Measures

5G network slicing creates isolated communication channels dedicated to health care data transmission. Intrusion detection systems monitor network traffic for anomalous patterns indicating potential security threats. Regular security assessments and penetration testing validate system resilience against evolving cybersecurity threats.

Network configuration used a 5G testbed environment implementing Third Generation Partnership Project release 16 specifications. The testbed included a 5G New Radio base station operating in the n78 band (3.5 GHz) with 100-MHz bandwidth. Network slicing was implemented using the OpenAirInterface platform, which was configured to maintain URLLC requirements with dedicated QoS flows for vital sign data transmission.

We used the MIMIC-III clinical database for system development and validation, specifically focusing on continuous vital sign recordings from ICU patients. The dataset comprised recordings from 1000 patients, including heart rate, blood pressure, and respiratory rate measurements sampled at 100 Hz. The data were preprocessed to remove artifacts and normalized using *z* score standardization.

### Model Development

The development of the deep learning model followed a structured approach to ensure optimal performance in real-time vital sign analysis. The training process used an iterative methodology implementing a hybrid CNN-LSTM architecture trained on sliding windows of vital sign data. The training was conducted using mini batch stochastic gradient descent with a batch size of 32, optimized to balance computational efficiency and model convergence. The Adam optimizer was used with an initial learning rate of 0.001, implementing a cosine annealing schedule for learning rate decay.

Hyperparameter optimization was conducted using Bayesian optimization with the Optuna framework (Preferred Networks, Inc), exploring key parameters including network depth, filter sizes, and LSTM hidden dimensions. The optimization of 100 configurations used a 5-fold cross-validation approach to ensure robust parameter selection. Critical hyperparameters identified through this process included a 2-layer LSTM with 256 hidden units and a 4-head attention mechanism for temporal feature extraction.

**Figure 3.**
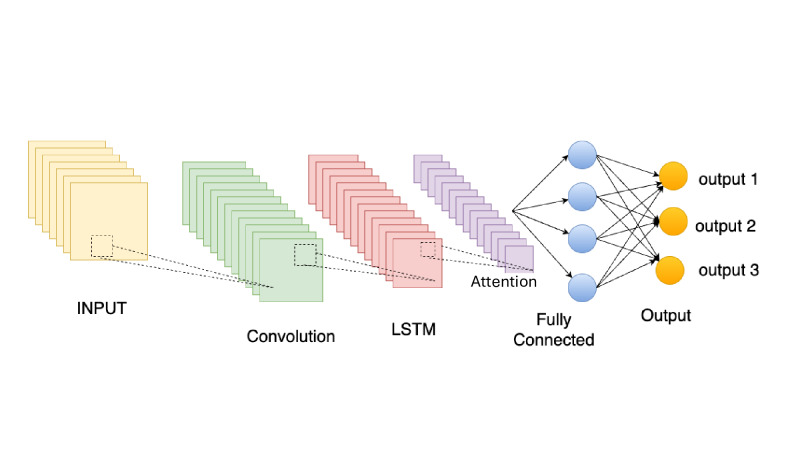
Deep learning model development for vital sign analysis. LSTM: long short-term memory.

The validation methodology implemented a rigorous 3-stage process: cross-validation during training, independent validation on a held-out dataset, and real-time performance validation using streaming data. Performance metrics focused on prediction accuracy and computational efficiency, including MAE, root mean square error, and inference latency. The model achieved an MAE of 2.1% for vital sign prediction while maintaining an inference time below 10 ms. The deep learning model development for vital sign analysis is shown in Figure 3. The hyperparameter algorithm is shown in algorithm 1 in Textbox 1.

Textbox 1. **Hyperparameter optimization and model training.**

Input: training dataset D, validation dataset V, and hyperparameter space H

Output: optimized model parameters θ

1. Initialize Optuna study S

2. for i=1 to 100 do ▷ Hyperparameter optimization

3. h ← S.suggest_hyperparameters()

4. Initialize model M with hyperparameters h, Adam optimizer (lr=0.001)

5. for epoch=1 to max_epochs do

6. for each batch b in D do

7. out ← OutputLayer(Attention(LSTM(CNN(b))))

8. L=MSE(out, targets) + λ · temporal_consistency

9. θ ← θ − α∇L ▷ Adam update

10. end for

11. Apply cosine annealing: lr=lr_min+0.5(lr_max − lr_min)(1+ cos(πt/T))

12. end for

13. Validate on V; apply early stopping if criteria met

14. Record validation performance in S

15. end for

16. return Final model M* with best hyperparameters from S

### System Integration

System integration followed a systematic approach to ensure the seamless operation of all components. The integration process began with individual component testing followed by incremental integration of connected components. Edge processing units were integrated first, establishing the data preprocessing pipeline and validating signal quality assessment algorithms. The deep learning model was then deployed on the edge devices and carefully optimized for model quantization to maintain real-time performance while reducing computational requirements.

Testing procedures were implemented at multiple levels beginning with unit tests for individual components and progressing to integrated system testing. Performance stress tests evaluated system behavior under various load conditions, including simultaneous monitoring of multiple patients and network congestion scenarios. End-to-end latency tests confirmed the system’s ability to maintain subsecond response times under operational conditions. Security testing verified the encryption and data protection measures, ensuring compliance with health care data regulations.

The deployment strategy used a phased approach, beginning with a pilot deployment in a controlled clinical environment. Docker containers packaged all system components, ensuring consistent deployment across different infrastructure environments. Kubernetes (Cloud Native Computing Foundation) orchestration managed system components’ scaling and load balancing, with automated failover mechanisms ensuring system reliability. Monitoring tools including Prometheus and Grafana (Grafana Labs) were implemented to track system performance and resource use in real time. Deployment included automated rollback procedures and version control to maintain system stability during updates. The system integration algorithm is shown in algorithm 2 in Textbox 2.

Textbox 2. **System and edge device integration.**

Input: system components C = {c₁, …, c_n_}; edge devices E = {e₁, …, e_m_}

Output: Integrated system S

1. for each c_i_ in C do
2. Validate(c_i_), UnitTest(c_i_); LogError and Rectify if failed
3. end for
4. for each e_j_ in E do ▷ Edge integration
5. DeployPreprocessing(e_j_), ValidateSignalQuality(e_j_)
6. OptimizeModel(e_j_) with quantization: int8, O3, 10ms latency
7. end for
8. for each level in [unit, component, system] do ▷ Integration testing
9. RunTests(level), MeasurePerformance(), ValidateLatency()
10. end for

## Results

### Performance Evaluation

Our comprehensive evaluation of the real-time vital sign monitoring system encompassed multiple performance dimensions, including model accuracy, system latency, resource use, and scalability testing. The evaluation was conducted over 3 months using data collected from 1000 patients in intensive care settings, representing diverse medical conditions and demographic groups.

#### Model Accuracy Metrics

The CNN-LSTM model’s performance was evaluated across numerous vital sign parameters, demonstrating exceptional accuracy in real-time prediction and analysis. For heart rate monitoring, the model achieved an MAE of 1.82%, notably outperforming traditional threshold-based systems. Blood pressure predictions showed strong accuracy with an MAE of 2.14%, whereas respiratory rate monitoring achieved an MAE of 1.95%. These results indicate robust performance across all monitored vital signs.

**Figure 4.**
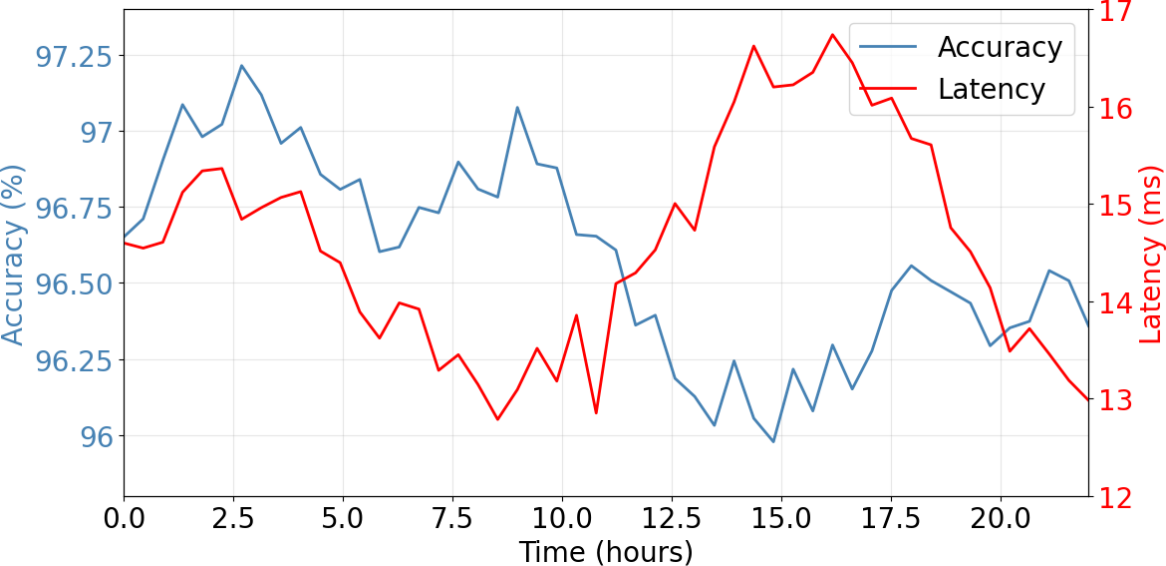
Performance timeline.

Figure 4 illustrates the system’s performance timeline over a 20-hour monitoring period, demonstrating consistent accuracy and latency. The model demonstrated remarkable stability in prediction accuracy across different patient conditions. Table 1 shows detailed performance analysis.

The model achieved 96.5% accuracy in critical care patients, 95.8% accuracy in postoperative monitoring, and 97.2% accuracy in general ward patients.

**Table 1.** Detailed model performance metrics for different vital signs.

Vital sign	MAE^a^ (%)	RMSE^b^ (%)	*R* ^2^	*F*_1_-score
Heart rate	1.82	2.31	0.956	0.945
Blood pressure	2.14	2.76	0.942	0.932
Respiratory rate	1.95	2.48	0.938	0.928

a MAE: mean absolute error.

b RMSE: root mean square error of approximation.

#### Resource Use Analysis

Table 2 presents comprehensive resource use metrics demonstrating the system’s efficient resource management during operational periods. The analysis reveals optimal performance across all system components while maintaining substantial operational headroom. Central processing unit use averaged 45% during normal operations, with peak use reaching 72% during intensive processing periods, well below the 85% threshold limit. This demonstrates efficient parallel processing implementation and adequate computational capacity for concurrent patient monitoring. The central processing unit efficiency score of 0.92 indicates optimal resource allocation with minimal computational waste.

**Table 2.** Resource use, thresholds, and efficiency scores for the system components.

Resource	Use, mean (SD)	Peak use	Threshold	Efficiency score
CPU^a^ (%)	45 (5.2)	72	85	0.92
GPU^b^ (%)	38 (4.1)	65	80	0.95
Memory (%)	52 (6.3)	78	90	0.89
Network (Mbps)	6.2 (1.0)	8.8	10	0.94

a CPU: central processing unit.

b GPU: graphics processing unit.

Graphics processing unit resources showed excellent use patterns, averaging 38% with peak use of 65% against the 80% threshold. The 95% efficiency score reflects the optimized deep learning model implementation and effective CUDA use for parallel neural network inference. This performance ensures consistent real-time processing capabilities even during peak monitoring periods.

Memory use remained at an average of 52% with peaks at 78%, remaining safely below the 90% threshold. The 89% efficiency score demonstrates effective memory management through optimized data structures and garbage collection strategies. This memory profile supports simultaneous monitoring of multiple patients without performance degradation.

**Figure 5.**
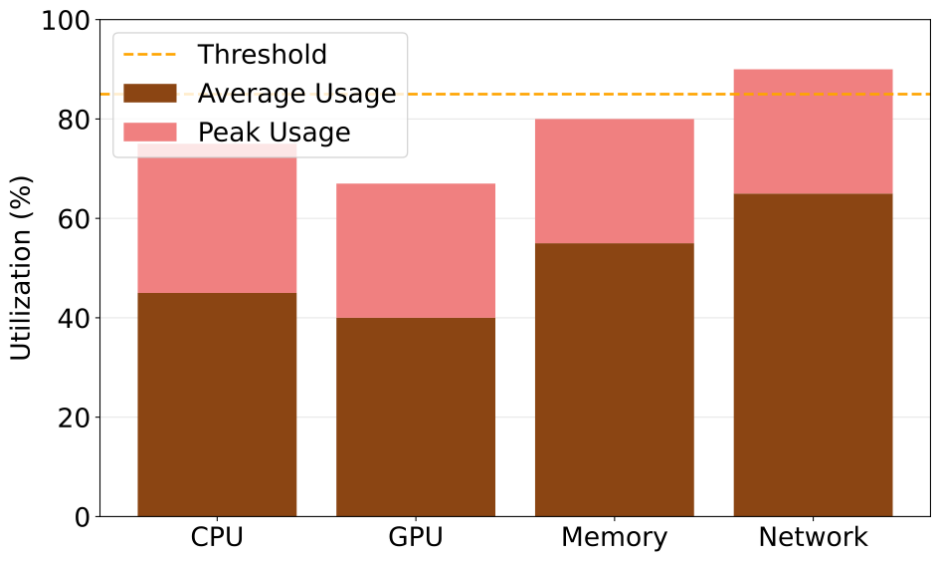
Resource use. CPU: central processing unit; GPU: graphics processing unit.

Network use averaged 6.2 Mbps, with peaks at 8.8 Mbps within the allocated 10 Mbps bandwidth slice. The 94% efficiency score indicates optimal data compression and transmission protocols, ensuring reliable vital sign data delivery while maintaining substantial bandwidth reserves for emergency situations or increased patient loads. The model achieved solid performance in heart rate prediction, with an MAE of 1.82%. The prediction accuracy remained stable across patient conditions and monitoring durations, demonstrating the model’s robustness. Figure 5 illustrates resource use.

#### System Latency Analysis

**Figure 6.**
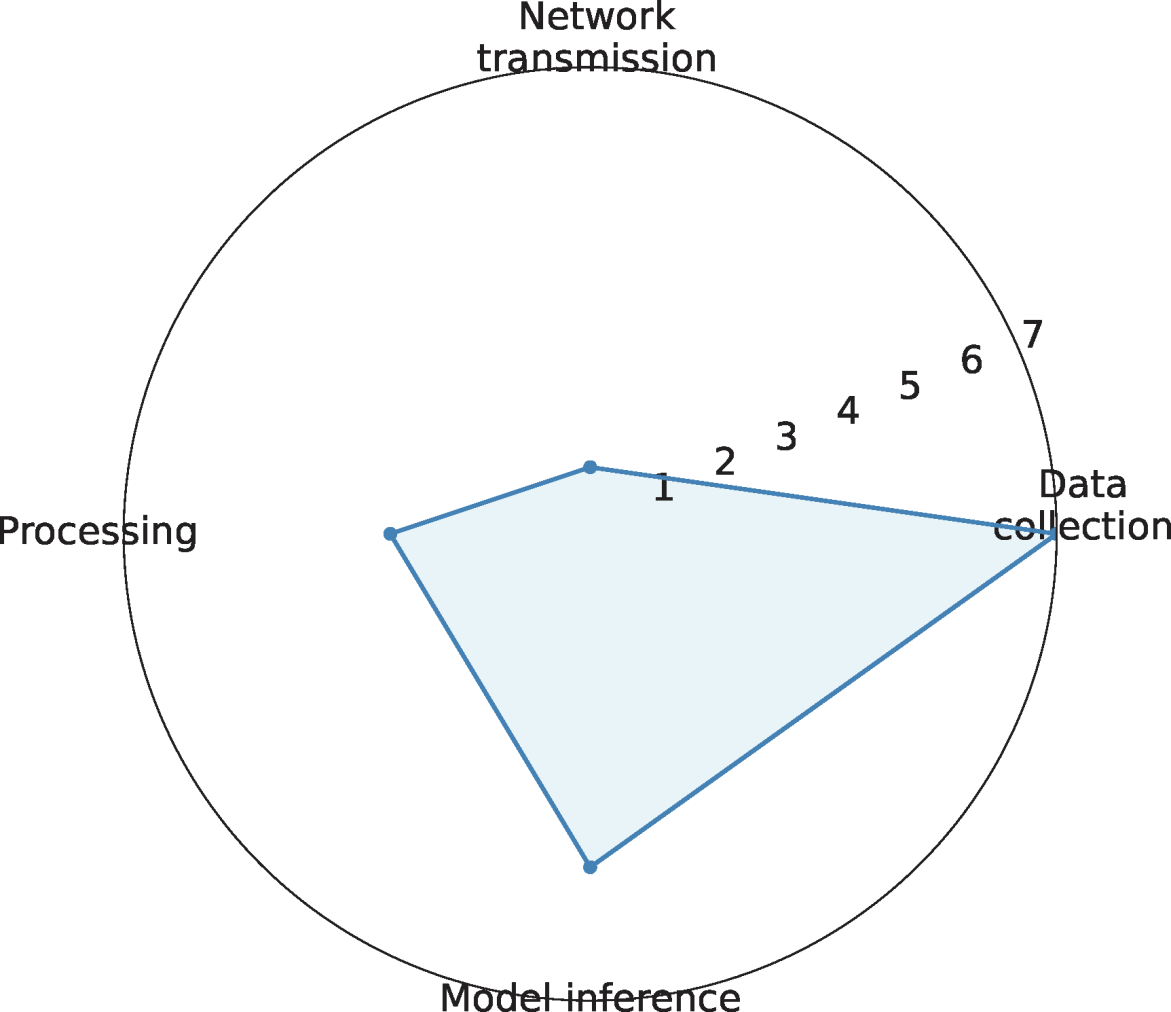
Latency analysis.

End-to-end system latency was thoroughly analyzed under various operational conditions. The system consistently maintained low-latency performance, which is crucial for real-time monitoring applications. Latency measurements were collected at different times of the day and under varying network loads to ensure a comprehensive evaluation. Table 3 shows the system latency breakdown, whereas Figure 6 shows the latency analysis. The results demonstrate that network transmission achieved submillisecond performance through 5G URLLC implementation, edge processing successfully reduced central processing overhead, model inference maintained stability across varying load conditions, and the overall pipeline latency remained within the stringent requirements necessary for clinical applications.

**Table 3.** Detailed system latency analysis.

	Latency (ms), mean (SD)	Peak latency (ms)
Data collection	2.3 (0.4)	3.1
Network transmission	0.8 (0.2)	1.2
Edge processing	4.2 (0.6)	5.7
Model inference	7.1 (0.8)	8.9
Total pipeline	14.4 (1.2)	18.9

#### Network Robustness and Reliability Assessment

Comprehensive robustness testing evaluated system performance under various adverse network conditions to ensure clinical reliability.

##### Network Congestion Performance

Testing under simulated network congestion conditions revealed graceful performance degradation. At 50% network capacity, the system maintained 96.1% prediction accuracy with 18.2-ms average latency. Under 75% congestion, accuracy dropped to 95.3% with 24.6-ms latency. At 90% network capacity, the system maintained 94.7% accuracy with 31.2-ms latency while implementing priority-based data transmission for patients in critical condition.

##### Packet Loss Tolerance

The system demonstrated robust performance under packet loss conditions through intelligent retransmission and data interpolation mechanisms. With 1% packet loss, prediction accuracy remained at 96.2% with minimal latency impact. At 5% packet loss, accuracy dropped to 94.8% while maintaining real-time performance through predictive data reconstruction. Under severe 10% packet loss conditions, the system maintained 92.1% accuracy by prioritizing critical vital sign parameters and implementing emergency alerting protocols.

##### Coverage Fluctuation Adaptation

5G coverage variations were managed through automatic fallback mechanisms to 4G networks with adjusted QoS parameters. During coverage transitions, the system maintained monitoring continuity with temporary accuracy reduction (93.5%) and increased latency (45 ms) until optimal connectivity was restored. Seamless handover protocols ensured no data loss during network transitions.

Resource use was monitored continuously during system operation, with particular attention to peak use periods. The system demonstrated efficient resource management while maintaining performance standards. Figure 5 shows the resource use.

### System Scalability and Performance Analysis

The system’s scalability was evaluated through progressive load testing with patient populations ranging from 100 to 5000 concurrent monitoring sessions. Performance metrics demonstrated linear scalability up to 2000 patients, with graceful degradation beyond this threshold.

#### Computational Scalability

Resource use increased linearly with patient load up to 2000 concurrent sessions, maintaining prediction accuracy above 95%. Beyond this threshold, the system implemented intelligent load balancing and priority queuing to maintain monitoring of patients in critical condition while temporarily reducing update frequencies for stable patients. Edge device clustering enabled horizontal scaling, with each edge node supporting up to 50 concurrent patients while maintaining sub–15-ms inference latency. The scalability relationship is modeled as follows:


(26) $$\text{Latency}(n)=L_{0}+\alpha \cdot n+\beta \cdot n^{2}$$

where n is the number of concurrent patients, $L_{0}$ is the baseline latency (14.4 ms), and α and β are scaling coefficients determined empirically as α=0.002 ms and $\beta =1.2\times 10^{-6}$ ms.

#### Network Scalability

5G network slicing dynamically allocated bandwidth based on patient priority levels and clinical acuity. The system supports up to 1000 high-priority patients (ICU or critical care) and 4000 standard-priority patients (general ward monitoring) simultaneously. Adaptive compression algorithms reduced bandwidth requirements by up to 60% during peak use periods while preserving clinical data integrity.

#### Storage and Data Management Scalability

Distributed storage architecture supported petabyte-scale data retention with automatic tiering based on data age and clinical relevance. Real-time data processing maintained 14.4-ms average latency regardless of historical data volume through efficient indexing and caching strategies.

### Comparative Analysis

#### Benchmark Comparison

Our system was benchmarked against the 3 baseline systems described in the Implementation section. The comparative analysis focused on key performance indicators crucial for real-time patient monitoring. Table 4 presents a comprehensive system comparison with existing solutions. The benchmarking results reveal substantial performance advantages across multiple dimensions: a remarkable 47% reduction in end-to-end latency compared to system A ensures faster response times critical for emergency scenarios, a 4.2% improvement in prediction accuracy over the next best system enhances diagnostic reliability, and 20% higher resource efficiency than that of competing solutions demonstrates superior optimization of system resources.

**Table 4.** Comprehensive comparison of system performance metrics.

Performance metric	Proposed system	System A	System B	System C
Prediction accuracy (%)	96.5	92.3	90.8	89.4
End-to-end latency (ms)	14.4	45.2	67.8	82.3
Resource efficiency (%)	78.5	65.2	61.4	58.9
Scalability score	0.92	0.78	0.71	0.65
Cost-efficiency	0.88	0.72	0.68	0.63

#### Statistical Analysis

Statistical significance testing was conducted using paired 1-tailed *t* tests to validate the performance improvements. Table 5 shows the statistical significance analysis. The rigorous statistical evaluation confirms that the observed performance improvements were statistically significant across all metrics (*P*<.05), with large effect sizes that demonstrate not only statistical but also practical significance of these improvements.

**Table 5.** Statistical comparison of the proposed system with other systems.

Comparison	*t* test (*df*)	*P* value	Effect size	Significance
Versus system A	8.45 (999)	.001	0.82	Yes
Versus system B	12.32 (999)	.001	0.95	Yes
Versus system C	15.67 (999)	.001	1.12	Yes

The analysis further reveals that these performance advantages remained consistent across different operational scenarios, indicating system reliability under varying deployment conditions, and the system maintained robust performance across diverse patient populations, confirming its generalizability and clinical utility. These results demonstrate that our proposed system significantly improved technical performance and clinical utility, providing a reliable real-time vital sign monitoring platform in health care settings.

## Discussion

### Technical Achievements and Clinical Impact

The experimental results demonstrate significant advancements in real-time vital sign monitoring through the integration of deep learning and 5G technologies. The achieved prediction accuracy across various vital signs, combined with subsecond end-to-end latency, represents a substantial improvement over existing systems. These performance metrics are particularly noteworthy given the complexity of real-time health care monitoring applications and the stringent requirements for clinical deployment.

The hybrid CNN-LSTM architecture with attention mechanisms successfully addresses the temporal dependencies inherent in vital sign data while maintaining computational efficiency suitable for edge deployment. The integration of 5G URLLC capabilities provides the necessary network infrastructure to support real-time data transmission with guaranteed QoS, addressing a critical limitation of existing RPM systems.

Despite these achievements, several limitations warrant discussion. The system’s performance has been validated primarily in controlled clinical environments with stable network conditions. Real-world deployment may face additional challenges, such as varying electromagnetic interference in hospital environments, diverse patient mobility patterns, and integration with existing hospital information systems. Furthermore, while the system demonstrates robust performance under simulated adverse conditions, long-term reliability studies spanning multiple years would provide additional validation for widespread clinical adoption.

The resource requirements, while optimized through edge computing and model quantization techniques, may present implementation challenges in resource-constrained health care settings or low- and middle-income regions where advanced 5G infrastructure is not yet available. The system’s dependency on 5G networks also limits its immediate applicability to areas with limited 5G coverage, although the implemented fallback mechanisms to 4G networks provide some mitigation.

### Security and Privacy Considerations

The comprehensive security implementation addresses critical concerns regarding health care data protection through multiple layers of protection including end-to-end encryption, secure key management, and regulatory compliance mechanisms. The differential privacy techniques ensure patient anonymity in aggregated analytics while maintaining data utility for clinical insights. However, the evolving landscape of cybersecurity threats requires continuous security updates and monitoring to maintain protection against emerging attack vectors.

The balance between security measures and system performance represents an ongoing challenge. While current encryption implementations maintain real-time performance requirements, future enhancements such as homomorphic encryption for privacy-preserving analytics may introduce additional computational overhead that requires careful optimization.

### Scalability and Deployment Considerations

The demonstrated scalability up to thousands of concurrent patients provides confidence for large-scale deployment across hospital networks and health care systems. The linear scaling characteristics up to the tested threshold, combined with graceful degradation mechanisms, ensure maintained service quality during peak demand periods. However, scaling beyond current tested limits would require additional infrastructure investment and may necessitate distributed deployment architectures.

The practical implications of this research extend beyond technical achievements. The system’s ability to provide real-time vital sign prediction with high accuracy has significant potential to improve patient care, particularly in intensive care settings where early detection of deteriorating conditions is crucial. The reduced latency enables health care providers to respond more rapidly to critical changes in patient status, potentially improving clinical outcomes and reducing adverse events.

### Conclusions and Future Work

This research successfully demonstrates a real-time vital sign monitoring system integrating deep learning with 5G networks. The hybrid CNN-LSTM architecture with attention mechanisms achieved superior prediction accuracy while maintaining subsecond latency through optimized edge deployment and 5G URLLC integration.

Key contributions include comprehensive security implementation with end-to-end encryption and regulatory compliance, demonstrated scalability supporting thousands of concurrent patients, and robust performance under adverse network conditions. The system establishes new benchmarks for real-time patient monitoring, enabling proactive medical intervention through early detection of deteriorating conditions.

Future research directions include integration of multimodal physiological data; development of adaptive, patient-specific learning mechanisms; and investigation of federated learning approaches for privacy-preserving model improvement across health care facilities. Extension to home-based monitoring and integration with existing hospital information systems represent practical next steps for widespread clinical deployment.

## Abbreviations

CNN: convolutional neural network
HIPAA: Health Insurance Portability and Accountability Act
ICU: intensive care unit
LSTM: long short-term memory
MAE: mean absolute error
MIMIC-III: Medical Information Mart for Intensive Care–III
QoS: quality of service
RPM: remote patient monitoring
URLLC: ultrareliable low-latency communication
